# Amikacin-loaded selenium nanoparticles improved antibacterial and antibiofilm activity of amikacin against bovine mastitis-causing *Staphylococcus aureus*

**DOI:** 10.1016/j.heliyon.2024.e41103

**Published:** 2024-12-11

**Authors:** Leila Asadpour, Maryam Alsadat Mehrbakhsh Bandari, Roozbeh Sojoudi Masouleh

**Affiliations:** Department of Biology, Rasht Branch, Islamic Azad University, Rasht, Iran

**Keywords:** Amikacin, Se nanoparticles, Drug resistance, *S. aureus*

## Abstract

**Background:**

Antibiotic resistance in various microorganisms has become one of the most serious health problems worldwide. The use of nanoparticles in combination with conventional antibiotics is one of the recent efforts to overcome these challenges. This study aims to synthesize and evaluate the possibility of using amikacin-loaded selenium nanoparticles as antibacterial agent against multidrug-resistant *Staphylococcus aureus*, that causes bovine mastitis.

**Methods:**

Selenium nanoparticles (SeNPs) were synthesized through chemical reduction of sodium selenite using L-cysteine. Loading of amikacin on selenium nanoparticles was done by mixing both in solution and confirmed by UV–Vis spectroscopy, XRD, SEM, and DLS. Antibacterial properties of obtained nanoparticles against *S. aureus* were determined using agar disc diffusion, broth micro dilution methods and also time-kill assay. Anti-biofilm properties of amikacin-loaded selenium nanoparticles was determined using microplate method and through determining the expression level of biofilm associated genes including *ica*A and *ica*D in *S. aureus* isolates treated with sub-MIC concentration of these nanoparticles by real-time PCR.

**Results:**

Synthetized SeNPs and amikacin-loaded selenium nanoparticles (SeNPs@AMK) exhibited spherical appearances and 80.4 % of Se° had a diameter of 120 nm. SeNPs and SeNPs@AMK exhibited antibacterial effects against *S. aureus* isolates at the range of 32–128 μg/mL and 1–32 μg/mL respectively. Dependent on concentration and the exposure time, bacterial killing was promoted by the SeNPs@AMK treatment. The use of SeNPs@AMK decreased the biofilm formation of the isolates by more than 50 % and also lead to down-regulation of *icaA* and *icaD* biofilm associated gene compared to the control.

**Conclusions:**

The results of this study suggest the antimicrobial properties of SeNPs and the reduction in the effective concentration of nanoparticle-loaded amikacin. Therefore, loading of antibiotics on the surface of nanoparticles may be used as a strategy to deal with the growing problem of drug resistance.

## 1 Introduction

Emergence and increase of antibiotic resistance in various microorganisms have become one of the most serious global health problems. Therefore, there is an urgent need to develop a new generation of antimicrobial agents to control infectious disease. Nanotechnology is one of the recent efforts in overcoming these challenges and nanoparticles are increasingly used in antimicrobial formulations [1,2]. Because of unique physicochemical and biological properties of nanoparticles such as their surface to mass ratio and their ability to adsorb and carry other components such as drugs, these particles are finding an increasing application in modern therapeutics [3]. The antibacterial effects of various metallic nanoparticles have been well documented and are significantly higher in comparison with metals [4]. One of the important features of metal nanoparticles is their ability to target different bacterial structures. Metal nanoparticles, such as certain selenium compounds, can influence the normal function of cell membrane permeability and also impair the process of cell respiration. Moreover, they can enter the cells, interfere with the function of proteins containing sulfur and phosphorus molecules, such as DNA, and destroy their effectiveness. A complex mechanism of metal nanoparticles, which are used to eliminate bacteria, virtually eliminates the possibility of resistance in bacteria [5,6].

Selenium is an essential micronutrient which regulates various physiological functions, such as cell differentiation and anti-inflammatory functions [7]. Furthermore, selenium is a powerful antioxidant, and plays an important role in the reduction of intracellular oxidative stress and chronic disease prevention. Consequently, selenium is an important factor in cellular metabolism and daily dietary intake of 53–60 μg Se/day is recommended [8]. Selenium nanoparticles (SeNPs) provide a stable and reliable means of therapeutic delivery in a wide spectrum of pathological conditions, such as cancer, neurodegenerative diseases, infections, and inflammation and also act as a redox-active nanomaterial, which play an important role in ROS regulation. In addition, the role of SeNPs in osteogenic maturation and their possible applications in bone treatment were investigated [7,9]. Furthermore, their antimicrobial and antibiofilm activity with minimal toxicity to animal cells is interestingly approved in their use as an antimicrobial agent [5,10].

Amikacin is one of the most important antibiotics that belongs to aminoglycosides and is characterized by its effect on most Gram-negative bacteria. Furthermore, amikacin displayed potent activity against Gram-positive bacteria including methicillin-sensitive and methicillin-resistant *Staphylococcus aureus.* The emergence of multidrug resistant pathogens and the persistent potency of amikacin have re-invigorated interest in this antibiotic [11]. However, due to its side effects, combination of amikacin with other antibiotics or non-antibiotics can extend its antibacterial spectrum and reduce its level of nephrotoxicity [12,13].

The use of antimicrobial nanoparticles in combination with conventional antibiotics is likely to reduce the use of high doses of drugs and plays a considerable role in reducing drug side effects, drug resistance and treatment costs. Theoretically speaking, compared with antibiotic molecules, carriers are kept longer in human body to achieve a long-term health [3]. In general, antibiotics bound with nanoparticles may be used in the future to treat infections, especially those that are drug resistant. Bovine mastitis is an important and costly disease which affects dairy industry worldwide. This disease is caused by several bacterial pathogens and is considered as one of the major causes of antibiotic use in dairy cows. *S. aureus* is one of the most prevalent etiological agents of contagious mastitis causing the most virulent and persistent mammary glands infections. Successful infection of this bacterium depends on production of several virulence factors including factors that facilitate bacterial adhesion and colonization [14,15]. Furthermore, resistance of *S. aureus* to antimicrobial agents is a well-documented challenge in dairy cows [16]. For this reason, there is a growing interest in finding new antimicrobial agents for combined therapy with antibiotics or using an alternative antimicrobial agent. This study aims to synthesize and evaluate the possibility of using selenium nanoparticles as antibacterial agents and carriers to enhance the effectiveness of amikacin against multidrug-resistant bovine mastitis-causing *S. aureus*.

## 2 Methods

### 2.1 Synthesis of selenium nanoparticles

Selenium nanoparticles (SeNPs) were synthesized through chemical reduction of sodium selenite using L-cysteine as described previously [17]. To confirm the synthesis of nanoparticles, UV–Visible spectroscopy, X-ray diffraction (XRD) spectroscopy, scanning electron microscopy (SEM), and dynamic light scattering (DLS) were used.

### 2.2 Loading of amikacin on selenium nanoparticles

First, 50 mg of selenium nanoparticles was poured into a 25 mL flask containing 10 mL distilled water, and was then placed in the ultrasonic bath for 10 min at medium speed until the nanoparticles were completely dispersed in the distilled water. Then, drops of amikacin solution at a concentration of 50 mg/L were added to the flask until the volume reached 25 mL. Subsequently, the mixture was incubated in a shaking incubator (150 rpm) for 24 h at 25 °C. The mixture was spin-dried at room temperature and the sediment was collected in another container. Loading of amikacin on selenium nanoparticles was approved by UV–Visible spectroscopy, XRD, SEM, and DLS.

### 2.3 Determining the percentage of amikacin-loaded Se nanoparticles (SeNPs@AMK)

For generating a amikacin calibration curve, various concentrations of amikacin solution were prepared in distilled water and the absorbance of each one was recorded by a spectrophotometer at a wavelength of 563 nm. In order to determine the concentrations of free amikacin in the solution, the supernatant was read by a spectrophotometer UV–Vis after centrifugation. Moreover, the concentration of drug in each sample was determined based on calibration curve. Finally, the concentration of free amikacin in the solution and the percentage of amikacin-loaded nanoparticle and drug loading efficiency (LE%) were estimated as described previously [15].

### 2.4 Test bacteria and antibiotic susceptibility profile

During 2023, the milk samples were taken from 150 cow with sub-clinical mastitis, in Guilan province, Northern Iran. Samples were taken following washing the teats, stripping of the first squirts of milk, and disinfection of the teat ends. The samples were aseptically collected into sterile tubes, placed in an icebox and transported to the laboratory. *S. aureus* isolates were identified according to previously described methods and criteria including investigation of growth characteristics on blood agar and Mannitol salt agar, Gram staining, catalase and coagulase tests [18].

The antibacterial resistance of test isolates was investigated according to the CLSI guideline (2020). An antibiotic disk (High Media, India), containing Cefoxitin (30 μg), Cephalexin (30 μg), Cephalothin (30 μg), Penicillin (10 μg), Amoxicillin (25 μg), Ampicillin (25 μg), Gentamicin (10 μg), Clindamycin (2 μg), Doxycycline (30 μg), Oxytetracycline (30 μg), Tetracycline (30 μg), Cotrimoxazole (23.75 μg), Erythromycin (15 μg), Enrofloxacin (5 μg), and Ciprofloxacin (5 μg), was used to determine the antibiotic sensitivity of test isolates. For further investigation, bacterial isolates, which showed resistance to at least three antimicrobial classes, were regarded as MDR strain. *S. aureus* ATCC 25923 was used as the standard strain in the experiments.

### 2.5 Phenotypic assessment of biofilm production in test bacteria

This assay was performed in microtiter plate. Briefly, standard overnight cultures (1.5 × 10^8^ CFU/mL) were diluted 100 folds in tryptic soy broth containing 1 % glucose. 200 μl of each culture dilution was transferred into individual wells of a 96-well flat-bottomed polystyrene plate and incubated overnight at 37 °C for 24 h. After incubation, the wells were rinsed three times with PBS to remove planktonic bacteria and subsequently fixed with methanol for 20 min. Thereafter, each well was stained with 200 μl of 0.02 % crystal violet and rinsed with distilled water for 5 min. Biofilm was quantitatively analyzed by adding 200 μl of 33 % glacial acetic acid to each well after drying the plates and reading their OD at 570 nm by ELISA reader. Strains were evaluated as strong (OD > 4 × ODc), moderate (4 × ODc > OD > 2 × ODc), weak (2 × ODc > OD > ODc), and negative biofilm (OD ≤ ODc) based on optical absorption. *Staphylococcus epidermidis* ATCC 35984 strains and *Staphylococcus epidermidis* ATCC 12228 strains were used as positive and negative biofilm formation controls, respectively [19].

### 2.6 Investigation of antibacterial properties of Se nanoparticles

#### 2.6.1 Determination of zone of inhibition

Sensitivity of test bacteria to selenium nanoparticles was determined using the standard disc diffusion method. Muller-Hinton agar plates were inoculated with 100 μl of standardized inoculum (1.5 × 10 ^8^ CFU/mL) of each bacterium and spread with sterile swabs. After that, the standard discs were coated with 100 μg of NPs and the plates were left at room temperature for 15 min to allow the diffusion of disc content into the agar. After incubation at 37 °C for 24 h, the inhibition zone around each well was measured in millimeters.

#### 2.6.2 Minimum inhibitory concentration (MIC)

Broth microdilution method was used to determine the minimum inhibitory concentration (MIC) of amikacin, SeNPs and SeNPs@AMK. For this purpose, 12 different concentrations including 0.5–2048 μg/mL sterile saline were prepared in Muller Hinton broth and 100 μl of standardized test organism suspension (1.5 × 10^7^ CFU/mL) was inoculated into each dilution. All tubes were incubated at 37 °C for 24 h. Then, the tube with lowest concentration of tested antibacterial agent, with no visible growth when compared with control, was considered as the MIC. The test tubes containing sterile saline and standard microbial suspension were treated as positive control, and tubes containing different concentrations of antibacterial agents and bacteria-free medium were treated as negative control. Each experiment was repeated twice [20].

#### 2.6.3 Time kill assay

The effectiveness of amikacin, SeNPs and SeNPs@AMK against clinical isolates of *S. aureus* was determined using the time-kill assay for different time intervals over a period of 24h. In these assays, overnight 5 × 10^5^ CFU*/*mL bacterial suspension was incubated in 96-well plate containing adjusted Mueller Hinton broth supplemented with above-mentioned antibacterial agents in separate wells. At time intervals (0, 2, 4, 8, 12, and 24 h) of incubation, aliquots were removed from each tube and diluted serially (1:10) with PBS for use in determination of cell viability in Muller-Hinton agar plates. Time–kill experiment was performed in triplicate [21].

### 2.7 Inhibition of biofilm development

For investigation of anti-biofilm activity of amikacin, SeNPs and SeNPs@AMK, the strong biofilm-forming *S. aureus* isolates were cultured into 96-well microplates as described above. Subsequently, sub-MIC concentrations of amikacin, SeNPs and SeNPs@AMK were added to each well and were incubated at 37 °C for 24 h. After incubation, rinsing of the plate, fixation with methanol and colorization of formed biofilm with crystal violet were considered. Finally, biofilm was quantitatively analyzed by adding glacial acetic acid to each well after drying the plates and reading their optical density (OD) as described above. The obtained OD values of each treated sample were compared with the control (20).

### 2.8 Biofilm eradication assay

The effects of SeNPs, AMK and SeNPs@AMK on preformed biofilms were investigated as described previously with some modifications [22,23]. In brief, an aliquot of 200 μL of bacterial suspension (1x10^6^ CFU/ml) in tryptic soy broth was dispensed into a 96-well flat bottom microplate. After 24 h, the media were discarded from the wells and to remove unattached cells, the plates were washed thoroughly with PBS. Thereafter, 100 μL of tryptic soy broth containing sub-MIC concentrations of SeNPs/AMK/SeNPs@AMK were added to the preformed biofilms. The plates were incubated for 24 h at 37 °C and then the media were discarded from the wells, 100 μL of PBS solution was added to each well containing biofilm and then biofilm cells were suspended by Vortex (30 s)- vigorous pipetting -Vortex (30 s). Subsequently, 10-fold dilutions were prepared from suspended biofilm and the recovery of *S. aureus* was quantitatively analyzed by colony forming units (CFU) assay by counting the colony number on tryptic soy agar plates. The culture without the antimicrobial served as positive control and Muller-Hinton broth without culture served as the negative control. The results have been reported as Log10 CFU/ml.

### 2.9 Gene expression analysis

The clinical strains of *S. aureus* were selected for molecular investigation based on their MDR phenotype and biofilm forming ability. PCR screening for the presence of selected biofilm-associated genes was done. Subsequently, after overnight treatment of fresh culture of selected *S. aureus* isolates with sub-MIC of SeNPs and SeNPs@AMK, RNA extraction and cDNA synthesis were performed using The High Pure RNA Isolation Kit (Bioneer Co., Germany) and cDNA synthesis kit (Yekta Tajhiz Azma, Iran), respectively. After that, quantitative real-time PCR test was performed in triplicate using icaA and icaD genes specific primers (Table 1) and the SYBR Premix Ex *Taq*II kit (Tli RNase H Plus), (Takara, Japan). The reaction was performed in the Rotor Gene Q (USA) according to the following program: initial degradation step at 95 °C for 30 s, 40 cycles at 95 °C for 5 s and 60 °C for 30 s. The *16S rRNA* gene was used as internal control and the relative expression of selected genes was calculated by ΔΔCт .

**Table 1:** Oligonucleotide primers used in this study.

Primer	Sequences 5′ to 3′	Annealing Temp.	Amplicon size (bp)	Ref.
*icaA*	GAGGTAAAGCCAACGCACTC CCTGTAACCGCACCAAGTTT	60	151	24
*icaD*	ACCCAACGCTAAAATCATCG GCGAAAATGCCCATAGTTTC	60	211	24
*16SrRNA*	GGGACCCGCACAAGCGGTGG GGGTTGCGCTCGTTGCGGGA	60	191	24

### 2.10 Cytotoxicity of SeNPs

The cytotoxicity of SeNPs against normal HEK-293 cell line was evaluated by examining mitochondrial dehydrogenases activity using 3-(4,5-dimethylthiazol-2-yl)-2,5-diphenyl-2H-tetrazolium bromide (MTT) assay kit (Yekta Tajhiz, Iran). Briefly, the cells were purchased from cell bank of Pasteur Institute of Iran and grown in DMEM medium supplemented with 10 % fetal bovine serum, 2 mM L-glutamine, penicillin (100 U/ml) and streptomycin (100 μg/mL). The cells were maintained at 37 °C with 5 % CO2 in a humidified CO2 incubator. Then, HEK-293 cell suspensions (1 × 10^4^ cells/wells) were seeded onto 96-well tissue culture plates and cells were treated with different concentrations (0–100 μg/mL) of SeNPs. After treatment, the 96-well plates were incubated for 24 h and subsequently, 10 μl of MTT dye was added into wells and incubated for 4 h. Finally, purple formazan crystals were dissolved in dimethyl sulfoxide (DMSO) and the optical density was read at 570 nm using ELISA reader. The cell toxicity was determined as previously described [24].

### 2.11 Statistical analysis

Experiments were performed in triplicate and data was analyzed by SPSS 18. One-way ANNOVA was used for the determination of statistical differences between the groups. P-value<0.05 was considered statistically significant.

## 3 Results

### 3.1 Characterization of selenium nanoparticles

**Fig. 1:**
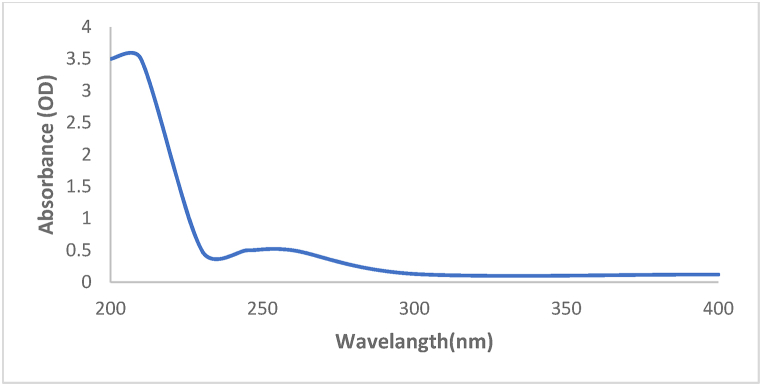
UV–Vis spectroscopy results of SeNPs.

**Fig. 2:**
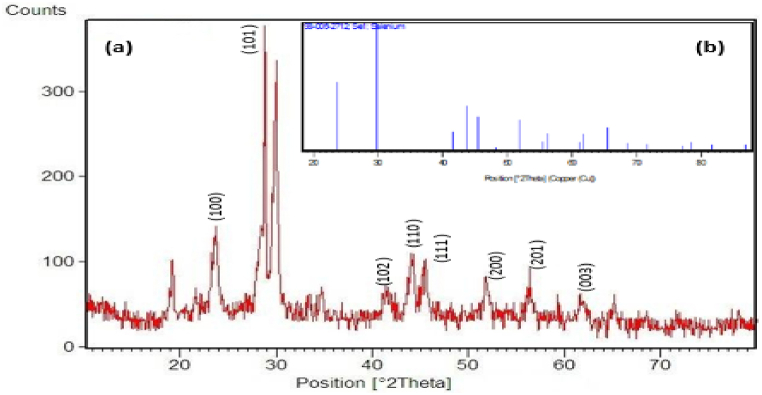
X-ray crystallography. (b) X-ray diffraction pattern of synthetized SeNPs, (b) X-ray crystallography peak of the standard sample (card 98-006-2712 JCPDS) of SeNPs.

**Fig. 3:**
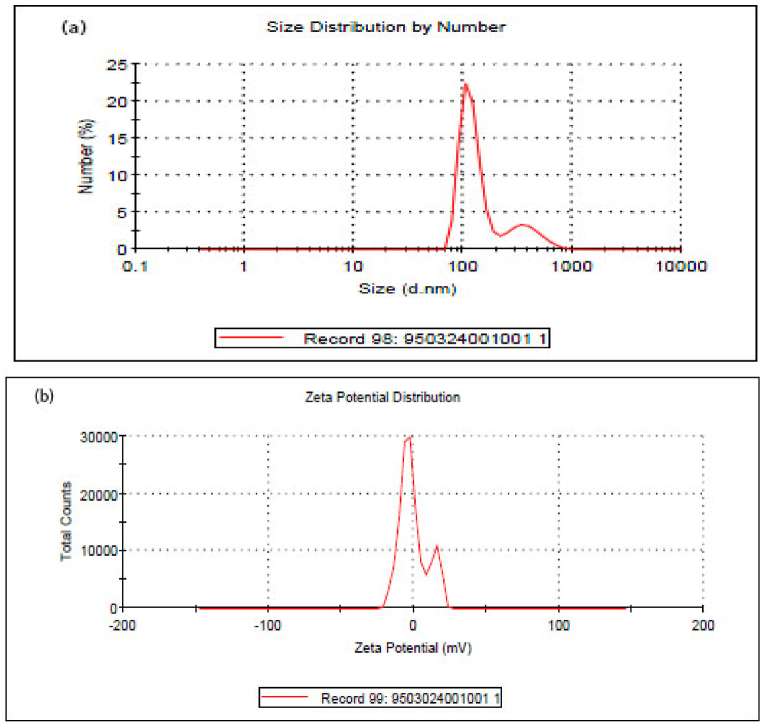
Particle size distribution graph obtained from diffractive light scattering analysis of SeNPs (a) and Zeta potential of SeNPs (b).

**Fig. 4:**
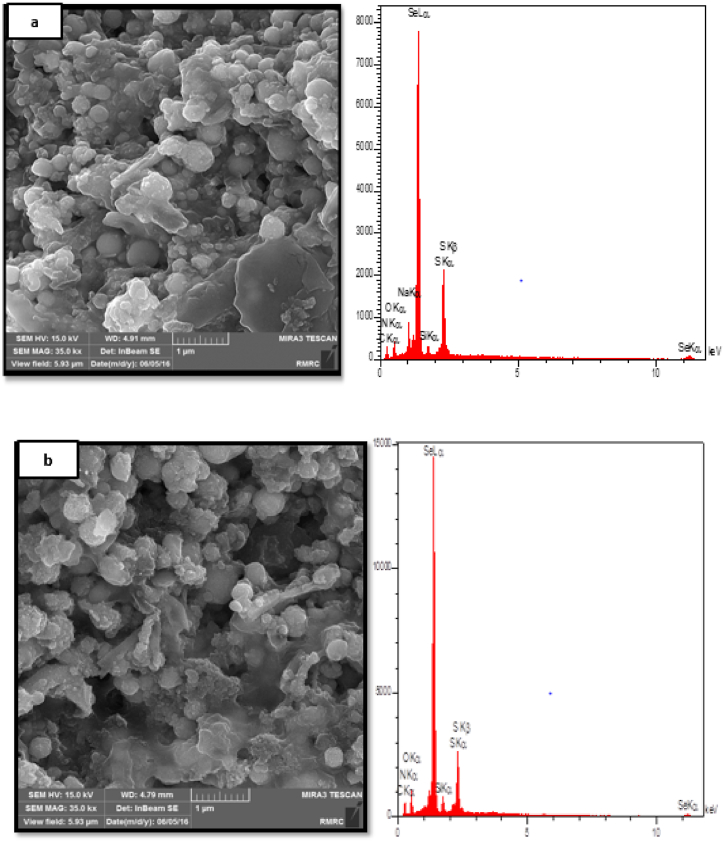
Scanning electron microscopy (FE-SEM, left) and (EDAX, right) of Dry powder of SeNPs (a) and amikacin-loaded SeNPs (b).

**Fig. 5:**
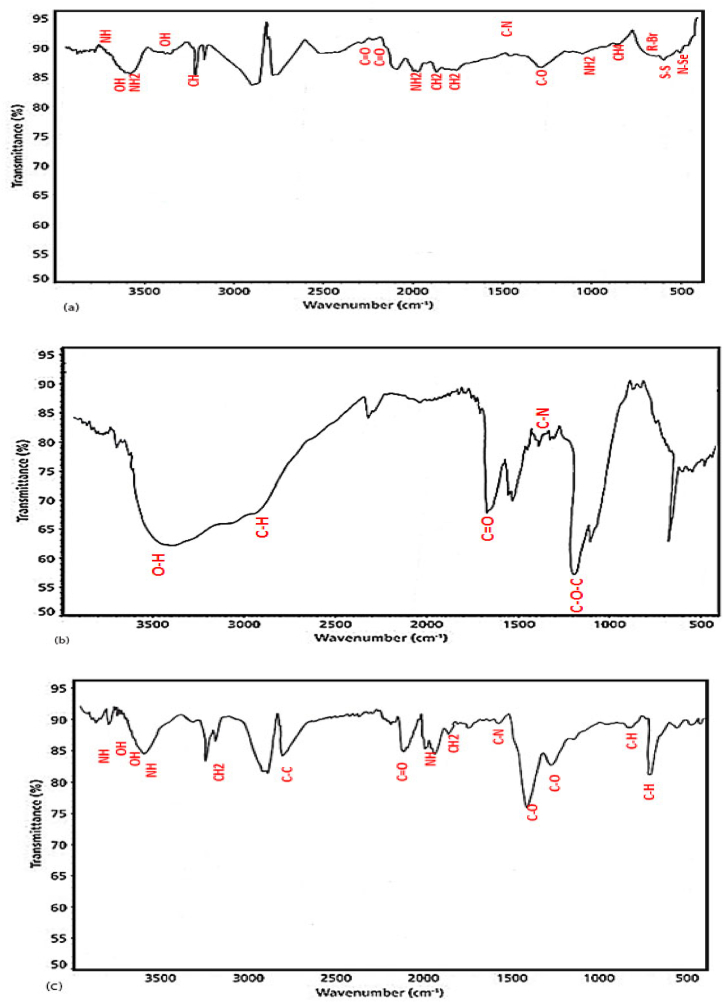
Infrared spectroscopy of SeNPs (a), amikacin (b) and amikacin-loaded SeNPs (c).

The reaction of chemical precipitation of sodium selenite with L-cysteine resulted in color change from white to orange, indicating deposition of SeNPs. The synthesis of nanoparticles was confirmed by UV–Vis spectroscopy, XRD spectroscopy, SEM and DLS analysis. UV–Vis spectroscopy of synthesized nanoparticles showed the highest level of light absorption within 218 nm wavelength, which matched with the results obtained from light absorption of SeNPs (Fig. 1). X-ray diffraction pattern of the crystalline structure of the synthesized nanoparticles was obtained at angles of 20°–80°. Two strong diffractions in the areas (100) and (101), and weak diffractions (102), (110), (111), (200), (201), and (003), which comply with the standard sample (cardJCPDS) with reference code 98-006-2712, confirm the correct synthesis of selenium nanoparticles (Fig. 2). Appearance of SeNPs was studied using scanning electron microscopy. The results of DLS in Fig. 3a show two size distributions of SeNPs. In the first summit, 80.4 % of particles have a diameter of 120 nm and 19.6 % of particles have a size of 387.1 nm. Zeta potential for SeNPs were recorded on average −14.2 mV (Fig. 3b). As shown in Fig. 4a (on the left), SeNPs exhibit spherical appearances. In Fig. 4a (on the right), the results of EDAX indicate the amount of selenium in both structures; the level of selenium is particularly high in SeNPs. Finally, the results of FTIR confirmed the stability of the chemical structure of the SeNPs (Fig. 5a).

### 3.2 Characterization of amikacin-loaded selenium nanoparticles

The results of infrared spectroscopy confirmed the stability of the chemical structure of the amikacin on selenium nanoparticles (Fig. 5). As shown in Fig. 4b (on the right), the results of EDAX indicate high levels of selenium in tested structures. After the loading of amikacin, the optical density of the supernatant obtained from the centrifuge was equal to 0.872. Consequently, using previously reported formula, concentrations of free amikacin in the solution, drug encapsulation efficiency (EE%), and percentage of amikacin-loaded nanoparticles have been estimated as 2.037, 89, and 44.5, respectively.

### 3.3 Test bacteria

A total number of 40 *S. aureus* were isolated from bovine sub-clinical mastitis samples. Preliminary screening was performed on these isolates. According to antimicrobial susceptibility test, 22 (55 %) isolates showed multidrug-resistant (MDR) phenotype. The biofilm-forming ability was detected in 25 (62.5 %) isolates; 6 isolates were strong biofilm formers, 12 isolates were moderate biofilm formers and 7 isolates were weak biofilm formers. Also, all MDR isolates were biofilm formers. For further investigation, 10 MDR biofilm former (6 strong and 4 moderate biofilm formers) *S. aureus* isolates were selected.

### 3.4 Antimicrobial activity of SeNPs and SeNPs@AMK

**Fig. 6:**
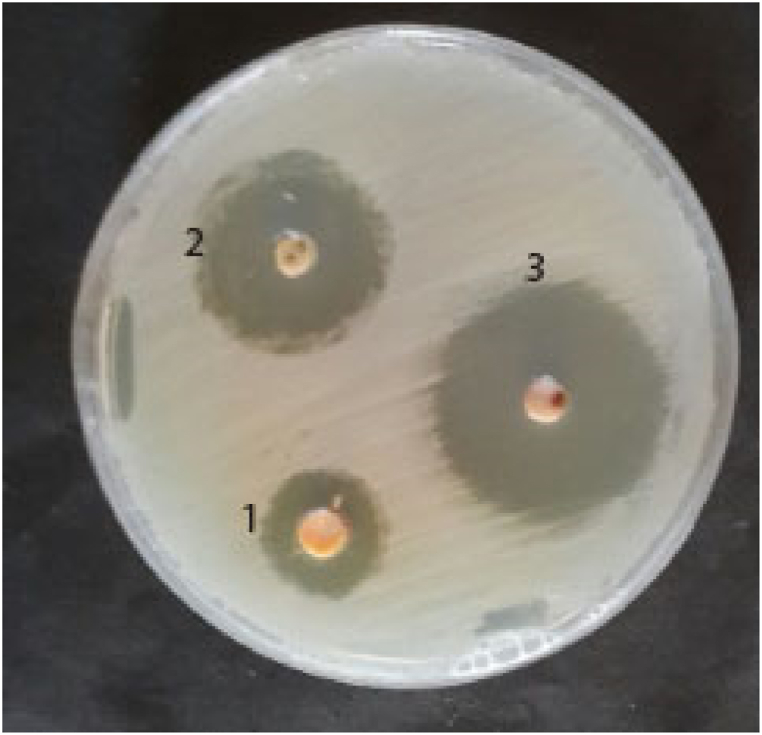
Zone of inhibition of growth of *S. aureus*. 1: 25 μg, 2: 50 μg, and 3:100 μg SeNPs@AMK.

SeNPs and SeNPs@AMK showed antimicrobial effects against all tested *S. aureus* isolates. As shown in Fig. 10, SeNPs and SeNPs@AMK inhibited growth of test bacteria. Diameter of zone of inhibition of 100 μg SeNPs and SeNPs@AMK ranged between 12-16 and 18–30 mm respectively (Fig. 6), and the MIC values ranged from 32 to 128 μg/mL and 1–32 μg/mL. Loading of amikacin at SeNPs improved its antibacterial effects with 2-16-fold decrease in MIC values. The data related to the MIC values of 10 selected bacteria are shown in Table 2.

**Table 2:** Evaluation of the antibacterial activity of SeNPs against *S. aureus* based on MIC (μg/ml).

No. of test bacteria	Amikacin	SeNPs	SeNPs@AMK
1	256	128	32
2	128	128	32
3	128	64	4
4	64	32	4
5	64	32	16
6	256	128	16
7	32	64	4
8	32	128	8
9	16	64	2
10	16	32	1

### 3.5 Inhibition of the biofilm development by SeNPs, AMK and SeNPs@AMK

**Fig. 7:**
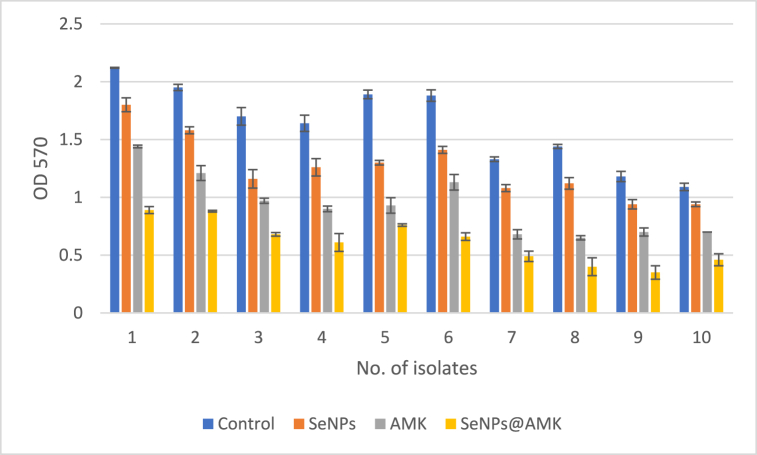
Anti-biofilm activity of SeNPs, AMK and SeNPs@AMK against clinical *S. aureus* isolates. Mean ± SD of OD570 of bacteria treated with sub-MIC concentration of test materials compared to control.

The microtiter plate method was used for evaluation of the anti-biofilm activity of SeNPs, AMK and SeNPs@AMK. The results are presented in Fig. 7. In all treatments with sub-MIC concentration, tested bacteria exhibited significantly lower biofilm formation compared to the control (P ≤ 0.05). SeNPs@AMK decreased the biofilm formation of the isolates between 55 and 72 % compared to the control.

### 3.6 The effects of SeNPs, AMK and SeNPs@AMK on pre-existing biofilms

**Fig. 8:**
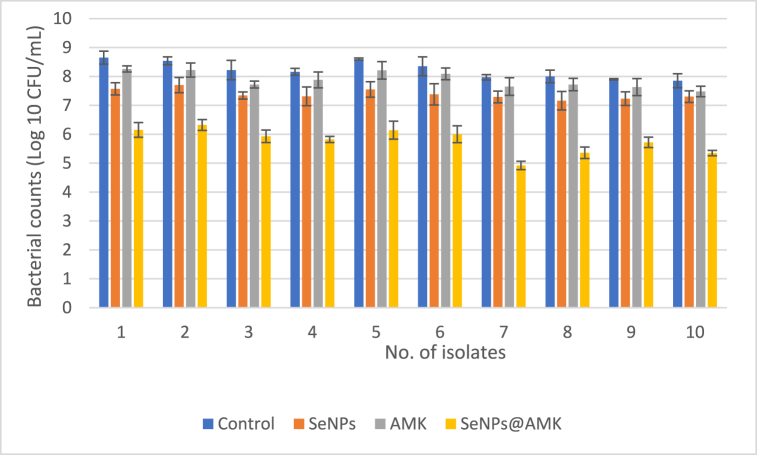
Pre-existing biofilm eradication activity of SeNPs, AMK and SeNPs@AMK against clinical *S. aureus* isolates. Mean ± SD of bacterial count (Log 10 CFU/mL) of biofilm-embedded bacteria treated with sub-MIC concentration of test materials compared to control.

The effects of AMK, SeNPs and SeNPs@AMK on biofilms were investigated based on the reduction in bacterial counts (Log10 CFU/mL0) in pre-existing biofilms treated with sub-MIC concentrations of tested anti-bacterial agents, as shown in Fig. 8. Compared to the control, no significant differences (P > 0.05) were observed in the number of viable cells for any of the isolates treated with free AMK. However, the lowest CFU was observed when *S. aureus* biofilm was treated with SeNPs@AMK where mean viable cell count decreased from 2.2 to 3 log10 CFU/mL compare to the controls.

### 3.7 Time-kill assay

**Fig. 9:**
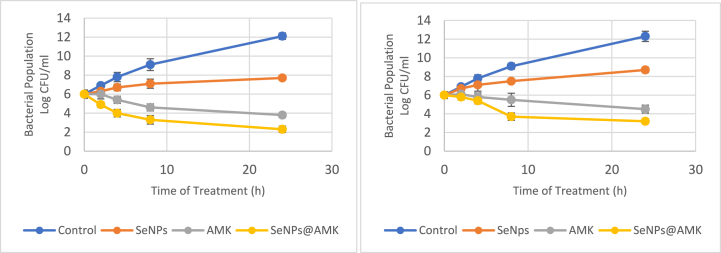
Bacterial killing kinetic (Mean ± SD of bacterial count (Log 10 CFU/mL) of SeNPs, AMK and SeNPs@AMK at 0.5 × MIC compared with control against selected clinical *S. aureus* isolates. Left: S3 isolate, Right: S8 isolate.

The bactericidal activity of SeNPs, AMK and SeNPs@AMK against the selected *S. aureus* isolates in terms of changes in the log10 CFU/mL of viable cells is shown in Fig. 9. In time-kill curve assay (Fig. 9), the control reached a bacterial population of more than 12 log10 CFU/mL after 24 h of incubation at 37 °C. A gradual reduction of the bacterial count at times between 0 and 24 h was observed with treatment at 0.5 × MIC of SeNPs and showed reduction of 4.4 and 3.4 log10 in two tested isolates after 24h of exposure. The number of viable *S. aureus* cells decreased more sharply by 0.5 × MIC of AMK and SeNPs@AMK and more than 8 units reduction in log10 of cells were detected after 24h in tested isolates treated with SeNPs@AMK.

**Fig. 10:**
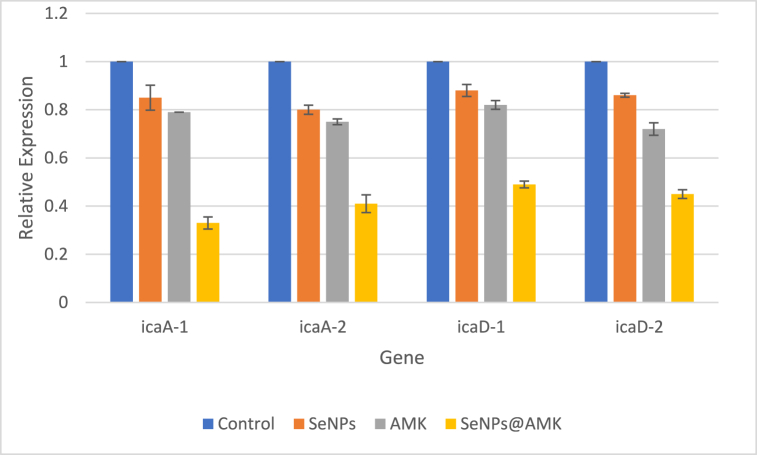
The effect of SeNPs, AMK and SeNPs@AMK on the expression of *ica*A and *ica*D genes. Mean ± SD of fold change in each gene expression level in bacteria treated with sub-MIC concentration of test materials compared to control.

### 3.8 Effect of SeNPs, AMK and SeNPs@AMK on the expression of *ica*A and *ica*D

The relative expression of *ica*A and *ica*D genes was evaluated in 3 selected *S. aureus* isolates treated with sub-MIC concentrations of SeNPs, AMK and SeNPs@AMK compared with untreated cells. These treatments significantly down-regulated the expression of *ica*A and *ica*D genes (p < 0.05) compared to the housekeeping gene (*16S rRNA* as control) in all test bacteria. Expression of *ica*A and *ica*D significantly decreased in the presence of sub-MIC concentrations of SeNPs, AMK and SeNPs@AMK compared with untreated cells. Moreover, when bacterial cells were exposed to sub-MIC concentrations of AMC/SeNPs, expression of *ica*A and *ica*D genes was decreased more than 50 % compared with control (Fig. 10).

### 3.9 Cytotoxicity of SeNPs

The cytotoxicity of different concentrations of SeNPs was tested on HEK-293 cells using MTT assay. The result was calculated as the percentage of viability of the cells cultured with six successive concentrations (3.12, 6.52, 12.5, 25, 50, 100 and 200 μg/mL) of SeNPs for 24 h. The viability of controls was set at 100 %. The obtained results revealed that SeNPs had no cytotoxic effect on cell viability at concentrations lower than 50 μg/mL. According to the results, more than 50 % of the cells treated with 100 μg/mL of SeNPs for 24h were viable.

## 4 Discussion

Mastitis is the most common disease leading to economic loss and the most important reason for antibiotic use in dairy industries. Multiple antibiotics such as penicillin, ampicillin, oxacillin, tetracycline, gentamycin, enrofloxacin, streptomycin, etc., which can be given by intra-mammary infusion or parenteral administration, have been exploited in current treatment of bovine mastitis [[25], [26], [27]].

*S. aureus* is one of the main causative agents of this infection in dairy cows worldwide. The persistent intracellular existence of *S. aureus*, its encapsulation in the mammary parenchyma and its ability to form biofilms helps it escape the immune response and hinders the penetration of many antimicrobials, resulting in a significant decrease in the efficacy of treatment for this infection. However, emergence of drug-resistant strains is a threatening viability of antibiotics for mastitis treatment. On the other hand, this proves that lower antibiotic use can result in decreased bacterial resistance [14,25,27].

Nanotechnology, however, is a rapidly growing field, offering the possibility of manufacturing new materials at the nanoscale level, with the formidable potential to revolutionize the agri-food sector by offering novel treatment options for prevalent and expensive illnesses such as bovine mastitis [28]. In the present study, antibacterial effects of SeNPs against bovine mastitis-causing *S. aureus* were evaluated. SeNPs showed significant antimicrobial effect on *S. aureus* isolates and inhibited the growth of this bacterium at concentrations ranging from 32 to 128 μg/mL. The inhibitory effect of SeNPs against *S. aureus* has been reported in several studies. Chudobova et al., reported that a concentration of 300 μg/mL of selenium nanoparticles can completely inhibit the growth of *S. aureus* [29]. Tran and Webster (2011) and Ramos et al. (2012) also confirmed the strong inhibitory effect of selenium nanoparticles against *S. aureus* [30,31].

Tran and Webster (2013) stated that 72 h exposure to selenium nanoparticles resulted in 90 % decrease in *S. aureus* [32]. In another study, SeNPs showed strong growth inhibition toward *S. aureus* at a concentration as low as 1 ppm [33].

Antibiotic-loaded nanocarriers offer significant advantages over conventional therapy, including targeted delivery to the disease site and the controlled release of drugs, which help maintain optimal drug concentration at the affected site [14]. In the present study, after loading amikacin on SeNPs, the minimum inhibitory concentration of drug against *S. aureus* decreased 2–10 times. In addition, a bacterial killing assay using sub-MIC concentration of SeNPs@AMK showed that the drug combination could enhance the inhibitory effect of amikacin on the growth of *S. aureus* strains and significantly reduced planktonic bacterial population in a time-dependent manner by more than 9 log10 CFU/mL. The selenium nanoparticles in connection with amikacin is likely to act as a carrier and enhances drug penetration through the bacterial membrane*,* which increases the antimicrobial activity of nanoparticle-loaded amikacin compared to the conventional forms of this drug. Furthermore, the controlled release of amikacin from SeNPs@AMK may occur during the treatment period which may increase efficacy and decrease the undesirable side effects [34]. Enhancing the antimicrobial properties of NPs bonded with antibiotics has been discussed in several studies. Enhanced antibacterial efficacy of amikacin in conjugation with silver nanoparticles was observed against *S. aureus* [35]. Also, it was found that the MIC of ciprofloxacin-loaded chitosan nanoparticles against *S. aureus* was lower than that of ciprofloxacin alone [27,36]. This finding supports our results which show that antibiotic-loaded nanoparticles can reduce the dose of antimicrobials.

In addition, in the present study, SeNPs and amikacin showed the ability to inhibit the formation of biofilms or eradicate pre-existing biofilms of mastitis-causing *S. aureus*. The biofilm inhibitory and biofilm eradicating potential of inorganic nanoparticles, including SeNPs gold NPs, silver NPs and copper NPs against mastitis-causing *S. aureus* have been revealed previously [37,38].

Also, aminoglycosides were previously reported to have antibiofilm activity at sub-MIC concentrations but are limited in their penetration through biofilm matrix [39,40]. We found that SeNPs@AMK can enhance biofilm inhibition and biofilm eradication potency compared to free amikacin. In treatment of pre-existing biofilms, a total reduction of 2.2–3 log10 CFU was achieved by treating biofilm cells of *S. aureus* with sub-MIC concentrations of SeNPs@AMK. However, for free AMK, only a reduction of 0.5 log10 CFU or even less was observed.

Moreover, when bacterial cells were exposed to sub-MIC concentrations of AMC/SeNPs, the expression of *ica*A and *ica*D biofilm associated genes decreased more than 50 % compared with control. Binding to nanoparticles can enhance aminoglycoside penetration in microbial biofilms and increase its anti-bacterial activity on biofilm cultures [34,41].

Overall, due to their low toxicity, great biocompatibility, strong capacity to scavenge free radicals, and effective antibacterial activity, there is a widespread interest in using SeNPs in the production of plants, poultry, fish, and livestock. Nevertheless, SeNPs showed low cytotoxicity, extensive Se applications and medical therapy with a selenium compound supplement in the current study, making it possible to contaminate the environment, which in turn could result in toxicity and create a risk for human health [42,43].

## 5 Conclusion

The results of this study demonstrated the enhanced antimicrobial properties of SeNPs@AMK including those associated with biofilms. SeNPs showed low cytotoxicity for mammalian cells. Therefore, loading of amikacin on the surface of these nanoparticles can be used as a strategy to deal with the growing problem of drug resistance and to reduce the abundant use of antibiotics in dairy industries. Consequently, nanoparticles can be suitable carriers for drug delivery systems, particularly in the treatment of localized infections caused by multidrug-resistant bacteria.

## CRediT authorship contribution statement

**Leila Asadpour:** Writing – review & editing, Supervision, Project administration, Methodology, Funding acquisition. **Maryam Alsadat Mehrbakhsh Bandari:** Methodology, Investigation, Funding acquisition. **Roozbeh Sojoudi Masouleh:** Writing – original draft.

## Consent for publication

The authors give the publisher the permission to publish this work.

## Data availability statement

Data will be made available on request.

## Funding

This research received no specific grant from any funding agency in the public, commercial, or not-for-profit sectors.

## Declaration of competing interest

The authors declare that they have no known competing financial interests or personal relationships that could have appeared to influence the work reported in this paper.
